# Predictive Tools for Severe Dengue Conforming to World Health Organization 2009 Criteria

**DOI:** 10.1371/journal.pntd.0002972

**Published:** 2014-07-10

**Authors:** Luis R. Carrasco, Yee Sin Leo, Alex R. Cook, Vernon J. Lee, Tun L. Thein, Chi Jong Go, David C. Lye

**Affiliations:** 1 Department of Biological Sciences, National University of Singapore, Singapore; 2 Communicable Disease Centre, Tan Tock Seng Hospital, Singapore; 3 Saw Swee Hock School of Public Health, National University of Singapore, Singapore; 4 Program in Health Services and Systems Research, Duke-NUS Graduate Medical School, Singapore; 5 Biodefence Centre, Ministry of Defence, Singapore, Singapore; 6 Centre for Health Services Research, National University of Singapore, Singapore; 7 Department of Medicine, Yong Loo Lin School of Medicine, National University of Singapore, Singapore; Tropical Medicine Institute Pedro Kourí (IPK), Cuba

## Abstract

**Background:**

Dengue causes 50 million infections per year, posing a large disease and economic burden in tropical and subtropical regions. Only a proportion of dengue cases require hospitalization, and predictive tools to triage dengue patients at greater risk of complications may optimize usage of limited healthcare resources. For severe dengue (SD), proposed by the World Health Organization (WHO) 2009 dengue guidelines, predictive tools are lacking.

**Methods:**

We undertook a retrospective study of adult dengue patients in Tan Tock Seng Hospital, Singapore, from 2006 to 2008. Demographic, clinical and laboratory variables at presentation from dengue polymerase chain reaction-positive and serology-positive patients were used to predict the development of SD after hospitalization using generalized linear models (GLMs).

**Principal findings:**

Predictive tools compatible with well-resourced and resource-limited settings – not requiring laboratory measurements – performed acceptably with optimism-corrected specificities of 29% and 27% respectively for 90% sensitivity. Higher risk of severe dengue (SD) was associated with female gender, lower than normal hematocrit level, abdominal distension, vomiting and fever on admission. Lower risk of SD was associated with more years of age (in a cohort with an interquartile range of 27–47 years of age), leucopenia and fever duration on admission. Among the warning signs proposed by WHO 2009, we found support for abdominal pain or tenderness and vomiting as predictors of combined forms of SD.

**Conclusions:**

The application of these predictive tools in the clinical setting may reduce unnecessary admissions by 19% allowing the allocation of scarce public health resources to patients according to the severity of outcomes.

## Author Summary

Dengue is a mosquito-borne tropical disease that poses a large economic and health burden to 40% of the world's population. Some dengue patients can evolve into a more severe form of the illness (severe dengue) and ideally should be hospitalized when this occurs. However, while only a small proportion of patients have severe dengue, many other patients are unnecessarily hospitalized. Being able to identify those patients at the highest risk of developing severe dengue can be very useful to reduce costs to hospitals and patients. In this study, we develop predictive tools to identify those patients that should be hospitalized. We find that some common features such as age, gender, fever duration, fever on admission, vomiting or abdominal distension can be good predictors of severe dengue. This is important because these features are easy to identify even in resource-limited settings where laboratory tests are not widely available.

## Introduction

After successful eradication programs of the dengue vector *Aedes* mosquitoes in Latin America in the 1940s, both disease and vector have resurged and disseminated globally since the late 1970s [1], posing substantial disease and economic burdens on tropical and subtropical regions [2], [3]. It is estimated that currently about 40% of the world's population live at risk of dengue infection and 50–100 million infections occur annually [4], [5].

In Singapore, an equatorial city state in South East Asia, intensive vector control programs initiated in the 1970s led to a substantial decline in dengue notifications [6]. Possibly as a result of reduced herd immunity [7], however, cyclical epidemics have occurred since the 1990s, leading to an estimated average annual economic impacts of US $115 million [8]. Dengue epidemics in Singapore lead to a strong demand for hospital beds and manpower, diverting resources for elective surgery and non-emergency admissions [9] and inflicting large public and private hospitalization costs (e.g. $50 US million in 2005 alone [8]). Despite this, only a small fraction of dengue infections in Singapore develop complications requiring hospitalization – for instance 6% and 21% of patients admitted for dengue into Tan Tock Seng hospital, Singapore, developed dengue hemorrhagic fever [DHF] in 2004 and 2007 respectively [10].

Under the widely adopted and longstanding World Health Organization (WHO) 1997 classification system, some dengue infections with complications were classified as dengue fever (DF), while not all patients classified as having DHF actually required hospitalization. Alternative classification systems were shown to be more sensitive than the WHO 1997 classification system in predicting severity of dengue in Indonesian children [11], showing the need to develop improved classification criteria. In addition, the classification into DF and DHF has been noted to lead to the false perception of low disease burden in the Americas [12]. The need to classify dengue cases according to their clinical severity [11]–[13] led the WHO to issue new classification criteria in 2009 for probable dengue with and without warning signs, and severe dengue (SD) [14]. The new classification criteria has been shown to facilitate dengue case management and surveillance [15] through better classification of severity [16], [17]. This revision makes it urgent to develop predictive tools conforming to the WHO 2009 dengue classification criteria to distinguish patients not likely to develop SD, for whom outpatient management may be appropriate, from those at greatest risk of SD and who require close monitoring and early therapeutic interventions in hospital.

Predictive tools to triage dengue patients into those that are most and least likely to develop complications allow better allocation of limited healthcare resources. By identifying risk factors at presentation that are predictive of complications after hospitalization, admissions may be reduced, alleviating pressure on the healthcare system and avoiding unnecessary costs to patients. Statistical procedures have been used to distinguish dengue severity predictors [18], [19] and predictive tools using logistic regression or classification and regression tree models have been developed to triage patients between dengue fever (DF) and other febrile illnesses [20]–[22], between DF and DHF patients [9], [23], between DF and dengue shock syndrome (DSS) or other severe outcomes [22], [24], [25], and, recently, between DF and subtypes of SD including plasma leakage [25] or internal bleeding and plasma leakage [26].

This study addresses the need to identify predictors for the new WHO 2009 dengue classification system by reporting the results of a retrospective analysis of adult dengue cases in Tan Tock Seng hospital, responsible for the treatment of approximately 40% of notified cases in Singapore [27]. The demographic, clinical and laboratory characteristics of confirmed adult dengue patients at presentation to hospital were employed to develop predictive tools of SD after hospital admission.

## Methods

**Figure 1:**
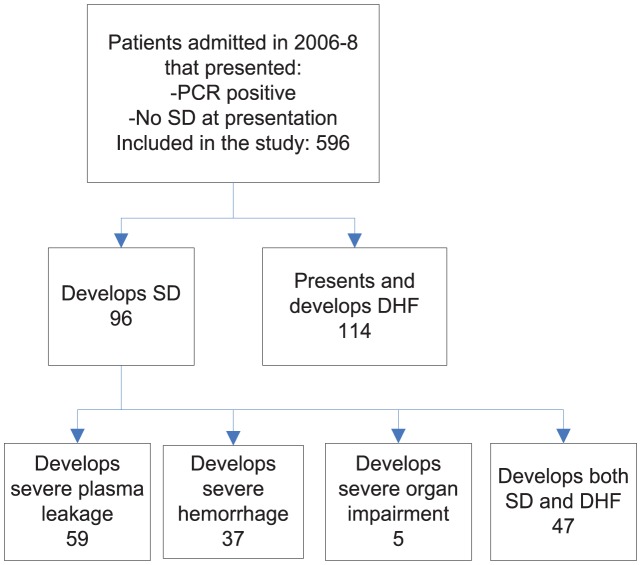
Study inclusion criteria. PCR: polymerase chain reaction, WHO: world health organization, DHF: dengue hemorrhagic fever, SD: severe dengue.

We conducted a retrospective cohort study in which we extracted from chart review demographic, epidemiological, co-morbidity, serial clinical and laboratory, radiological, treatment and outcome data from all confirmed adult dengue patients treated using a standardized dengue care path in the Department of Infectious Diseases, Tan Tock Seng Hospital, from 2006 to 2008. Prior to admission, patients were mainly referred from primary care centers where the standard is to monitor with full blood count, give symptomatic medication for fever, pain, nausea or diarrhea, and tell the patients to rest and drink copious fluid. Two groups of laboratory-confirmed dengue cases were considered: (i) had positive dengue polymerase chain reaction (PCR) (definite dengue) [28]; (ii) were positive to dengue immunoglobulin-M (IgM) or immunoglobulin-G (IgG) (probable dengue) (Dengue Duo IgM & IgG Rapid Strip, Panbio Diagnostic, Queensland, Australia). Patients who were diagnosed with severe dengue at the time of hospital presentation were excluded from further analysis; patients without SD but with warning signs were included (Figure 1). SD cases fulfill at least one of three criteria: (1) severe plasma leakage: associated with shock (tachycardia >100 or narrow pulse pressure <20 or blood pressure <90 mmHg) or respiratory distress, (2) severe hemorrhage: gastrointestinal tract bleeding such as haemetemesis or melaena or bleeding per rectum, pack cell transfusion or blood transfusion requirement or menorrhagia (3) severe organ impairment: defined as aspartate or alanine transaminase ≥1000units/l, acute renal impairment (creatinine ×2 upper limit of normal for age and sex (based on modification of diet in renal disease equation for Glomerular filtration rate = 75 ml/min) or baseline estimated from minimum creatinine recorded), encephalopathy and myocarditis [14]. In addition, the WHO proposed warning signs suggestive of high risk for SD were recorded: abdominal pain or tenderness, persistent vomiting (vomiting during two or more consecutive days), clinical fluid accumulation, mucosal bleed, lethargy or restlessness, hepatomegaly and concurrent increase in hematocrit and rapid decrease in platelet count (hematocrit change > = 20% concurrent with platelet <50×10^9^/l occurring in the same day) [14].

Variables presenting missing data in a proportion greater than 10% were removed from the analysis. Those variables with fewer missing data had their missing values replaced by an imputed value being the mean over all individuals in the study. For dichotomous variables, missing values were imputed by noes, the assumption being that if the sign or symptom were present, it would have been noted by the attending clinician. Variables that were not clinically relevant to severe dengue (e.g. runny nose, sore throat) or that conveyed similar information or information that was a linear combination of other variables were further removed from the analysis. Variables corresponded to values at presentation with the exception of hematocrit change > = 20% and drop in platelet count was obtained comparing the value at presentation with values during the course of the disease. In total, 66 explanatory variables extracted from chart review at presentation were employed and classified in groups: demographic and epidemiological characteristics; co-morbidities; symptoms and signs at presentation; laboratory results at presentation; and miscellaneous variables (see the Supporting Information Table S1 for the list of variables considered). Co-morbidities were scored for: congestive heart failure, cerebrovascular disease, chronic pulmonary disease, liver disease, diabetes, hemiplegia, renal disease, malignancy and AIDS. The scores were combined into the Charlson score and two variables indicating whether the individual had any comorbidity and whether individuals had Charlson score greater than three were respectively created. The large number of variables allowed us to minimize potential confounders while retaining variables that were clinically relevant. Prior to the analysis, we further tested the presence of collinear variables using variance inflation factors. Variables with a variance inflation factor greater than 3 were removed from the analysis stepwise using the package car in the R environment [29].

Predictive tools for SD development after presentation were constructed using logistic regression or its equivalent generalized linear models (GLM) with a logit link function and binomial errors [30]. Logistic regression or the GLMs used relate a group of explanatory variables to the log of the odds of a patient developing severe dengue versus not developing it. The GLMs were constructed and simplified using a stepwise procedure that started with the full model and removed one variable at a time using a chi-square test [31], [32].

**Figure 2:**
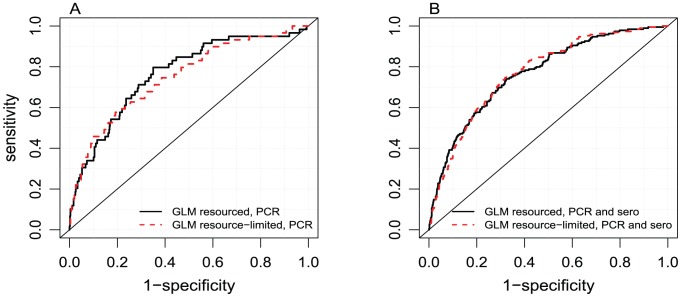
Comparison of the receiver operator characteristic curve in resourced and resource-limited settings for the GLM models fitted to predict SD in PCR confirmed cases (A) and PCR and serology confirmed cases (B).

The receiver operating characteristic (ROC) curve, the area under the ROC curve, sensitivity and specificity of the GLM models were estimated. Because our aim was to minimize the number of SD cases triaged for outpatient care, while allowing practitioners to decide on the tradeoff between sensitivity and specificity, we reported the specificity of the models given high sensitivity rates of 90%, 95% and 100% (see Figure 2, for a comparison of the ROC curves). Given the large number of potential predictors and to avoid the risk of overfitting or finding relationships by chance alone, we corrected the estimates of sensitivity and specificity for optimism—decrease in model performance in new patients compared to those in the sample studied [33]. The bootstrapping method was used to create samples with replacements from the dataset. A new model repeating all the stepwise model simplification process was constructed. The performance of the model on the prediction of the bootstrapped sample and the original sample was estimated. The optimism estimate was the difference between both performances and deducted to the original estimates of sensitivity and specificity [33]. 100 simulations were conducted per model to obtain stable estimates of optimism. In addition, the models were further validated using 5-fold validation by which the data are partitioned in 5 subsets, 4 subsets – the training dataset – are used to fit the GLM model and then employed to predict the remaining subset of the data – validation dataset. The procedure is then repeated changing the subset used as the validation dataset. The 5-fold validation process was repeated 1000 times and the mean of the root mean squared prediction error (RMSPE) was estimated for each model. The estimation of RMSPE was also subjected to optimism correction. Lower RMSPE indicated a better performance in the prediction of new cases and robustness of the model to potential associations found by chance alone. The R statistical environment was used for all analyses [34].

This study received ethical approval from the Domain Specific Review Board, National Healthcare Group, Singapore (DSRB-E/08/567) and all data analyzed were anonymized.

### Analyses

Dengue cases were analyzed with and without laboratory information so that appropriate tools could be developed for both well-resourced and resource-limited settings. GLMs were fitted to six combinations of data and variables:

- GLM fitted to only PCR confirmed cases including laboratory variables to predict any form of SD.
- GLM fitted to only PCR confirmed cases excluding laboratory variables to predict any form of SD.
- GLM fitted to PCR and serology confirmed cases including laboratory variables to predict any form of SD.
- GLM fitted to PCR and serology confirmed cases excluding laboratory variables to predict any form of SD.
- GLM fitted to only PCR confirmed cases including laboratory variables to predict severe hemorrhage.
- GLM fitted to only PCR confirmed cases including laboratory variables to predict severe plasma leakage.

## Results

Our cohort comprised 596 PCR-positive confirmed dengue cases of whom 96 [16.1%] developed SD (Figure 1). After hospital admission, severe plasma leakage developed in 59 (10%), severe hemorrhage in 37 (6%) and severe organ impairment in 5 (0.7%). Severe plasma leakage and severe hemorrhage occurred simultaneously only in 3 cases, showing little overlap between these two forms of SD. Severe organ impairment, on the other hand, occurred jointly with severe bleeding or severe plasma leakage in 4 cases out of 5.

The median age was 37 years (interquartile range [IQR], 27–43 years) and 450 individuals (75%) were male. Any co-morbidity was noted in 20% and only 5 patients had Charlson's co-morbidity score ≥3. Warning signs were present on admission to hospital in 65% of the patients. The median duration of illness prior to hospital presentation was 4.2 days (IQR, 3–5 days). Intravenous fluid therapy was administered to 91%, platelet transfusion to 9% of the patients and blood transfusion to two patients. Two patients were admitted to intensive care and one patient died. The median length of hospitalization was 5 days (IQR, 4–6 days).

For descriptive purposes we performed univariate tests, however, these results have no predictive power and should not be over interpreted because they do not control for the rest of variables and should not be used to triage patients. We also present results of the final outcomes of hospitalization (e.g. length of hospitalization, total platelet transfusion) that cannot be used to predict SD. Univariate analysis indicated that, at presentation, the cases that subsequently developed SD were more likely to be female and to present with shorter duration of fever and more likely to be febrile at presentation (Table 1). Fewer had leukopenia, and more had hypoproteinemia (Table 1). These patients received significantly more platelet transfusion during the course of the disease and stayed longer in hospital.

**Table 1:** Comparison of selected demographic, clinical and laboratory explanatory variables, and patient outcome data between dengue PCR positive patients with and without SD.

*Demographic and epidemiological*	Non-SD	SD	p-value
Age (years)	37 (27–44)	36 (25–42)	0.18
**Female**	**97/500 (20%)**	**49/96 (51%)**	**<0.001**
Any co-morbidity	99/500 (19%)	20/96 (21%)	0.86
*Selected clinical features*			
**Duration of fever at presentation**	**4.3 (3–5)**	**3.9 (3–5)**	**0.033**
**Fever**	**362/500 (72%)**	**85/96 (88%)**	**<0.001**
Headache	303/500 (61%)	58/96 (60%)	0.81
Eye pain	20/500 (4%)	2/96 (2%)	0.52
Myalgia	394/500 (79%)	75/96 (78%)	0.95
Arthralgia	114/500 (23%)	19/96 (20%)	0.53
Rash	165/500 (33%)	26/96 (27%)	0.13
Any bleeding	193/500 (39%)	35/96 (36%)	0.32
**Leukopenia**	**402/500 (80%)**	**71/**96 **(74%)**	**0.04**
*Plasma leakage*			
Hematocrit change ≥20%	30/500 (6%)	4/96 (4%)	0.37
Pleural effusion or ascites	0/500 (0%)	0/96 (0%)	na
**Hypoproteinemia**	**196/500 (39%)**	**52/**96 **(54%)**	**0.004**
*Warning signs*			
Abdominal pain or tenderness	146/500 (29%)	28/96 (29%)	0.77
**Persistent vomiting**	**228/500 (46%)**	**62/96 (64%)**	**0.01**
Clinical fluid accumulation	4/500 (0.8%)	0/96 (0%)	0.36
Mucosal bleeding	95/500 (19%)	20/96 (21%)	0.83
Lethargy or restlessness	176/500 (35%)	31/96 (32%)	0.33
Hepatomegaly	9/500 (2%)	1/96 (1%)	0.94
Rapid rise in hematocrit and drop in platelet	17/500 (3%)	2/96 (2%)	0.42
*Other clinical and laboratory data*			
Intensive care admission	1/500 (0.2%)	1/96 (1%)	na
Blood transfusion	0/500 (0%)	2/96 (2%)	na
**Platelet transfusion**	**38/500 (8%)**	**19/**96 **(20%)**	**<0.001**
**Duration hospital stay (days)**	**5 (4–6)**	**6 (5–6)**	**<0.001**
Death	0/500 (0%)	1/96 (1%)	na

Continuous data are summarized by median values and interquartile range, dichotomous by numbers and proportions. P-values are determined using the Wilcoxon's tests for continuous variables and Chi-square test for dichotomous variables. Covariates with statistically significant differences (p-value<0.05) in median or proportions are highlighted in bold font. Note: the univariate tests are only descriptive and should not be used for the triaging of patients.

### GLM models in well-resourced settings applied to PCR confirmed cases

We fitted GLMs to all types of SD combined as a single explanatory variable. The GLMs obtained optimism-corrected 29% specificity for 90% sensitivity (21% specificity for 95% sensitivity) for well-resourced settings with clinical and laboratory variables (Table 2, Figure 2).

**Table 2:** Sensitivity and specificity of the GLMs for the prediction of SD using only PCR-positive dengue.

Setting compatibility	Data	Response variable	Specificity (Sens = 1)	Specificity (Sens = 0.95)	Specificity (Sens = 0.9)	RMSPE
resourced	PCR	Any SD	0.01 (0)	0.26 (0.21)	0.41 (0.29)	2.57 (5.34)
resource-limited	PCR	Any SD	0.05 (0)	0.21 (0.15)	0.37 (0.27)	3.15 (5.19)
resourced	PCR and serology	Any SD	0.08 (0)	0.30 (0.25)	0.40 (0.30)	3.02 (4.25)
resource-limited	PCR and serology	Any SD	0.10 (0)	0.36 (0.30)	0.40 (0.30)	2.84 (4.03)
resourced	PCR	SH	0.27 (0)	0.42 (0.35)	0.66 (0.47)	5.47 (13.81)
resourced	PCR	SPL	0.13 (0)	0.20 (0.14)	0.30 (0.20)	5.22 (10.06)

SH: severe hemorrhage; SPL: severe plasma leakage. Sens: sensitivity for which each specificity estimate corresponds. Values between parentheses are optimism-corrected estimates. RMSPE: root mean squared prediction error.

The GLM to predict SD in well-resourced settings contained 8 explanatory variables (Table 3). Higher risk of SD development was associated with female gender, fever on admission, abdominal distension and vomiting (Table 3). Lower risk of SD development was associated with more years or age, leucopenia and normal levels (36.1–44.3% for females and 40.7–50.3% for males) of the minimum reading for hematocrit (Table 3). For instance, the mean of minimum hematocrit levels in females that did and did not develop SD was 35.5 and 36.6% and for males it was 39.4 and 41.6% respectively.

**Table 3:** Results of the GLM fitting to PCR confirmed cases for the identification of SD in well-resourced settings.

	Estimate	Odds ratio	95% CI	p-value
Intercept	3.47	-	-	-
**Age (years)**	−0.03	0.97	0.95–0.99	0.01
**Leucopenia**	−0.62	0.54	0.30–0.96	0.03
**Minimum hematocrit level (%)**	−0.11	0.90	0.84–0.96	0.00
**Gender female**	1.15	3.17	1.76–5.75	0.00
**Fever duration (days)**	−0.26	0.77	0.62–0.93	0.01
**Fever on admission**	1.17	3.23	1.65–6.92	0.00
**Vomiting**	0.56	1.75	1.07–2.88	0.03
**Abdominal distension**	3.01	20.24	1.66–485.33	0.02

The predictive equation yields odds (*ODD*) that are transformed into probability (*p*) by: *p* = *e^ODD^*/(*e^ODD^*+1). Patients with *p* greater than 0.0765, 0.0535 should be hospitalized to obtain sensitivities of 0.9, 0.95 and the corresponding specificities of 0.29, 0.21 respectively.

### GLM models in resource-limited settings applied to PCR confirmed cases

Because laboratory tests are not available in some resource-limited settings, we constructed GLMs that excluded laboratory variables. The resulting drop in the specificity of the predictive tools was small and they even obtained lower optimism-corrected prediction errors (RMSPE of 5.19 compared to 5.34 in the case of GLMs for resourced settings, Table 2). This could indicate that this model had fewer tendencies to overfitting than a model using laboratory variables and made them comparable to tools reliant on laboratory information. The GLMs obtained 27% specificity for 90% sensitivity (15% specificity for 95% sensitivity. Table 2, Figure 2, implying a reduction of 8–12% in specificity).

The GLM to predict SD in resource-limited settings contained 6 explanatory variables (Table 4). Similar to the GLM fitted with laboratory data, higher risk of SD development was associated with abdominal distension, female gender, vomiting and fever on admission; lower risk was associated to older age and fever duration on admission (Table 4).

**Table 4:** Results of the GLM fitting to PCR confirmed cases for the identification of SD without laboratory information.

	Estimate	Odds ratio	95% CI	p-value
**Intercept**	−1.15	-	-	-
**Age (years)**	−0.03	0.97	0.95–0.99	0.00
**Female**	1.63	5.10	3.08–8.52	0.00
**Fever duration (days)**	−0.32	0.73	0.59–0.89	0.00
**Fever on admission**	1.16	3.18	1.64–6.72	0.00
**Vomiting**	0.51	1.66	1.02–2.71	0.04
**Abdominal distension**	3.82	45.74	3.00–1158.85	0.01

The predictive equation yields odds (*ODD*) that are transformed into probability (*p*) by: *p* = *e^ODD^*/(*e^ODD^*+1). Patients with *p* greater than 0.0753, 0.0476 should be hospitalized to obtain sensitivities of 0.9, 0.95 and the corresponding specificities of 0.27, 0.15 respectively.

### GLM models applied to PCR and serology confirmed cases

When fitted to data combining PCR and serology confirmed cases, the GLMs obtained increases in specificity compared to GLMs fitted only to PCR data: e.g. 30% optimism corrected specificity for 90% sensitivity for resourced and resource-limited settings (Table 2, Figure 2, increase of 1–3% in specificity with respect to resourced and resource-limited settings using only PCR confirmed data). These models presented an optimism-corrected predictive error lower than the models fitted to PCR data alone (RMSPE of 4.03–4.25 versus 5.19–5.34).

The GLMs to predict SD in resourced and resource-limited settings contained 9 and 8 explanatory variables respectively (Tables S2 and S3). In both models, higher risk of SD development was associated with abdominal distension, female gender, breathlessness, vomiting and fever on admission (Table 4). More years of age and longer fever duration on admission were associated with lower risk of SD development.

### Predictors of subtypes of SD: severe plasma leakage and severe hemorrhage

We fitted GLMs using severe plasma leakage and severe hemorrhage as dependent variables. We could not find robust GLMs for severe organ impairment because of the small number of cases (5 cases). Fitting to severe hemorrhage led to higher specificity than fitting to the development of SD in general (Tables 2). Despite their increase in specificity, GLMs fitted to single forms of SD would not be able to reduce hospital admissions substantially further: cases with lower risk of severe plasma leakage may be at risk of severe hemorrhage and vice-versa, impairing the identification of cases with overall low risk of SD that could be managed as outpatients. These models presented however the highest optimism-corrected predictive errors compared to those predicting SD in general, indicating low robustness in the prediction of new cases (Table 2).

Fitting to specific forms of SD was nonetheless useful for comparison with other studies and to increase our understanding of the development of specific forms of SD. We found 6 predictors of severe hemorrhage (Table S4). Less years of age and female gender were common predictors with development of SD in general. High serum urea levels and hemoglobin count were new predictors of severe hemorrhage. Concurrent increase in hematocrit and rapid decrease in platelet count is a warning sign proposed by WHO for SD which was found to be good predictor of severe hemorrhage (Table S4). The prediction of severe plasma leakage presented 7 predictors. Shorter fever duration, vomiting, abdominal distension and fever on admission were predictors shared with any form of SD (Table 3 compared to S5).

### Warning signs as predictors of severe dengue

Several warning signs proposed by WHO 2009 for SD were identified as significant predictors. Abdominal distension – which was a good predictor of SD development – and could be considered related to abdominal pain and vomiting that was significant in models combining PCR and serology data. Concurrent increase in hematocrit and rapid decrease in platelet were found to be statistically significant in predicting severe hemorrhage and severe plasma leakage [14]. Vomiting and abdominal distension were good predictors of severe plasma leakage (Table S5).

### Potential cost-saving

We used a mean length of hospitalization of 5 days from our data, a mean cost per hospitalized case per day of US 2010 $431 [35], a mean cost per ambulatory visit of $62.1 and 4.33 visits per episode [8]. We estimated that the savings per case managed as outpatient in a well-resourced setting like Singapore would be $1886. Using the optimism-corrected sensitivity and specificity of the GLM in a well-resourced setting fitted to PRC confirmed data, reduction in admissions of 19% could be obtained. In the case of Tan Tock Seng hospital in Singapore, incorporating the diagnostic testing costs also to all probable dengue cases from 2006 to 2008 (1920 cases), a total cost to patients of US $0.69 million could be avoided ($0.23 million per year). Extrapolating to national level using the proportion of admissions at Tan Tock Seng hospital, US $0.58 million could be averted annually in Singapore.

## Discussion

The new disease severity classification for dengue issued by the WHO in 2009 has necessitated a reassessment of clinical care, management and diagnosis of dengue in many parts of the world. We developed predictive tools to optimize the triage of adult dengue patients in Singapore according to the new guidelines. In the Singapore context, applying these tools would reduce unnecessary admissions by 19%, alleviating demand on scarce hospital beds and healthcare costs to patients. However, these results are based on adopting models with a sensitivity rate of 90%, i.e. 10% of the SD patients would not be hospitalized at presentation. For sensitivities of 95%, reductions of 16% could be instead obtained.

It is difficult to compare our current results with studies dealing with the prediction of DHF and DSS according to WHO 1997 classification, given the different WHO 2009 classification that we employ. Good predictors of DHF were not necessarily good predictors of severe dengue. Carlos *et al.* [19] found a relationship between DHF and restlessness, nose bleeding, abdominal pain and low platelet count but, with the exception of abdominal pain, related to abdominal distension, we did not find these as significant predictors of severe dengue. Lee *et al.* [23] found that clinical bleeding, serum urea, and serum total protein were good predictors of DHF. Our results concur with respect to serum urea (only in the case of prediction of severe hemorrhage).

It was noteworthy that some of the warning signs (abdominal distension, vomiting) proposed by WHO were prognostic of SD when predicted jointly and for specific forms of SD. The apparent low predictive power of other WHO warning signs might respond to the application of intravenous fluid. This application might have eventually prevented the development of SD, particularly shock due to severe plasma leakage and could confound the results. The predictive models are hence trained to predict SD under current fluid therapy conditions and might underperform in resource-limited settings where fluid therapy might not be so prevalent. Another potential reason for the low performance of warning signs is that warning signs tend to appear on the day of defervescence. Given the objective of creating triaging tools, the data were collected at presentation and thus warning signs had not yet been observed in some cases. We further note that two warning signs with slightly different definitions to those provided by the WHO were employed: vomiting during two or more consecutive days and hematocrit change greater than 20%. Using the flexibility to exercise clinical judgments of the WHO guidelines, these warning signs were chosen because they have been shown to be good predictors in the local context [36], [37].

Comparison with other studies focusing on dengue severity prediction, on the other hand, showed some common features. Comparison was not straightforward because several of the studies, contrasting with ours, were based on univariate analyses or specific types of severity only. In relation to abdominal distension as a strong predictor, SD was associated with abdominal pain [22], [25], [26]. We identified below normal serum hematocrit as good predictor of SD. This has been found in previous studies in Thai children [24] and agrees with the prediction of internal bleeding [26] and severe outcomes [22] respectively. Further research would however be needed to unveil the actual mechanisms that could explain this result.

In predicting plasma leakage alone, we found that frequent vomiting was associated with plasma leakage, which agrees with previous studies [24], [26], [38], [39]. In contrast with our results, plasma leakage has been associated with older patients and male gender [25] but we could not find such association in our severe plasma leakage specific model. We found however that SD in general was associated with female gender and less years of age. The differences between studies might be due to the further inclusion of laboratory variables and by controlling for the presence of any co-morbidity and by using the Charlson score. This could avoid confounding effects with age in our study. Another potential reason for differences is that our cohort is relatively young (interquartile age of 27–43 years old and only 7% of individuals above 60 years old). Hence the lower risk of SD effect with an extra year of age that we find is mostly representative of young adults and middle age individuals. A precautionary approach should be taken when using the model to predict SD for patients above, for instance, 60 years old as this would involve extrapolating beyond an age for which the cohort was not as representative. We thus contend that further investigation on the relationship between age and SD outcome are thus necessary, preferably with cohorts mainly composed of old age individuals.

Interestingly, the variables necessary to predict any form of SD without laboratory variables were easier to measure than those aimed at predicting specific forms of SD such as plasma leakage. These results might derive from the heterogeneity of the different SD types. The predictive tools identify those less exclusive variables that are common to severe plasma leakage, severe bleeding and severe organ impairment. Among these variables, fever on admission, fever duration and vomiting are easy to measure and can lead to rapid and cost-effective triaging of patients.

Our study has some limitations. (i) Due to its retrospective nature, it is difficult to ascertain the homogeneity in the level of training of doctors and nurses with regards to the identification of clinical variables that could be subjective (e.g. persistent vomiting, abdominal pain) and how these could be applied to other settings. We have attempted to describe the clinical variables used to minimize ambiguities but different training in other hospitals could lead to variations in their identification. (ii) The GLMs were developed in an overwhelmingly adult cohort, and their performance in pediatric patients more common to other countries in the region needs to be validated. (iii) Not including other febrile illnesses in our study prevented the models from developing discriminatory power against them. This is both one of the strengths of the analysis, because we obtain highly fine-tuned predictive tools for SD, and a caveat, because our predictive tools may need to be supported by dengue diagnostic tests, which might not be easily available in resource-limited settings. Alternatively, published predictive tools to discriminate between DF and other febrile illnesses [20]–[22] can be used as a prior filter to the application of our predictive tools. (iv) Although the dengue epidemics in Singapore in 2006 were predominantly caused by dengue serotype 1, the epidemics in 2007 and 2008 were predominantly caused by dengue serotype 2 [10]. We controlled for serotype via a proxy (year), which was not statistically significant, but further studies in other settings would be needed to verify the validity of our predictive tools for epidemics driven by dengue serotypes 3 and 4. (v) Although serological tests for dengue are less accurate than PCR [28], for comparison we decided to include the results both with PCR and serology tests and PCR combined. Choosing only PCR confirmed cases was initially not preferred as there could be a risk that PCR confirmed cases favored patients that present earlier in the course of the disease and were febrile. If the bias had existed, we would expect fever on admission to be a good predictor in the case of only PCR confirmed cases and not in PCR and serology confirmed cases. This bias appears not relevant and consequently our results on fever on admission are also robust given that fever on admission is significant for both types of datasets (PCR and PCR and serology combined). We note that models fitted to both datasets agreed in a large number of predictors such as age, gender, fever duration, fever on admission , vomiting or abdominal distension. (vi) Our analysis does not control for the exact day of illness. Future research could focus on the prediction of SD one day ahead using the time series of the values of the explanatory variables through time. Although such models would not allow for patient triaging at admission, they will be useful to understand the development of SD and allow for the preparation of treatment at the hospital setting.

The easily implementable predictive tools developed in this study do not require laboratory-based variables and may be beneficial in resource-limited settings, where pressure on scarce health resources is especially high. In addition, no considerable reductions in predictive power were observed in resource-limited tools with respect to resourced tools. Their application will allow redistribution of resources to those patients and conditions that need them more urgently without incurring extra screening costs. In conclusion, we have shown a combination of clinical and laboratory data performed well in identifying potential patients with severe dengue.

## Supporting Information

Table S1 Explanatory variables considered at presentation.
(DOCX)

Table S2 Results of the GLM fitting to PCR and serology confirmed cases for the identification of SD in well-resourced settings.
(DOCX)

Table S3 Results of the GLM fitting to PCR and serology confirmed cases for the identification of SD without laboratory information.
(DOCX)

Table S4 Results of the GLM fitting to PCR confirmed cases for the prediction of SD due to severe hemorrhage.
(DOCX)

Table S5 Results of the GLM fitting to PCR confirmed cases for the prediction of SD development due to severe plasma leakage.
(DOCX)
