# Supplementary material for: Predictive Tools for Severe Dengue Conforming to World Health Organization 2009 Criteria
Source: PLoS Negl Trop Dis. 2014 Jul 10;8(7):e2972. doi: 10.1371/journal.pntd.0002972 (PMC4091876; doi:10.1371/journal.pntd.0002972)
Supplement: Table S1 — Explanatory variables considered at presentation. (DOCX) [file pntd.0002972.s001.docx]

Table S1. Explanatory variables considered at presentation

| **Explanatory variable** | **Extended definition** |
| --- | --- |
| Age, years |  |
| Abdominal pain |  |
| Serum alanine aminotransferase, unit/l |  |
| Ethnicity (Indian, Malay, others, Chinese as a baseline) |  |
| Fever duration in days |  |
| Abnormal vision |  |
| Respiratory rate per minute |  |
| Serum aspartate aminotransferase, unit/l |  |
| Charlson’s co-morbidity score ≥ 3 |  |
| Any co-morbidity |  |
| Aches and pains |  |
| Fever on admission |  |
| Serum creatinine, μmol/l |  |
| Pitt bacteremia score ≥ 4 |  |
| Lung dullness |  |
| Arthralgia |  |
| Temperature ⁰C |  |
| Serum potassium, mmol/l |  |
| Past history of dengue infection |  |
| Gender |  |
| Pulse per minute |  |
| Back pain |  |
| Serum sodium mmol/l |  |
| Year of admission 2007, 2008, 2006 as a baseline |  |
| Narrow pulse pressure <20 mmHg |  |
| Breathlessness |  |
| Serum urea, mmol/l |  |
| Hemorrhagic manifestation |  |
| Chest pain |  |
| Systolic blood pressure, mmHg |  |
| Basophil proportion, % |  |
| Rapid rise in hematocrit and drop in platelet | hematocrit change >=20% concurrent with platelet < 50x10^9^/l occurring in the same day |
| Postural hypotension > 20mmHg |  |
| Chills or rigors |  |
| Hematocrit change ≥20% |  |
| Abdominal distension |  |
| Hematocrit, % |  |
| Hypoproteinemia | Total protein < 63 g/l |
| Abdominal tenderness |  |
| Diarrhea |  |
| Hemoglobin, g/l |  |
| Hypotension | Systolic blood pressure < 90 mmHg |
| Heart murmur |  |
| Dizziness or giddiness |  |
| Lymphocyte proportion, % |  |
| Lethargy |  |
| Hepatomegaly | Palpable liver below costal margin, radiological hepatomegaly |
| Leukopenia | Total white cell count < 3.6⋅10^9^/l |
| Eye pain |  |
| Neutrophil proportion, % |  |
| Lung crackles |  |
| Fever |  |
| Platelet count, ∙10^9^/l |  |
| Serum hematocrit |  |
| Lymphadenopathy |  |
| Headache |  |
| Leukocyte count, ∙10^9^/l |  |
| Mucosal bleeding |  |
| Petechiae |  |
| Myalgia |  |
| Rash |  |
| Nausea |  |
| Red eyes |  |
| Positive pain score |  |
| Rash |  |
| Vomiting |  |
