# Supplementary material for: Predictive Tools for Severe Dengue Conforming to World Health Organization 2009 Criteria
Source: PLoS Negl Trop Dis. 2014 Jul 10;8(7):e2972. doi: 10.1371/journal.pntd.0002972 (PMC4091876; doi:10.1371/journal.pntd.0002972)
Supplement: Table S2 — Results of the GLM fitting to PCR and serology confirmed cases for the identification of SD in well-resourced settings. (DOCX) [file pntd.0002972.s002.docx]

Table S2. Results of the GLM fitting to PCR and serology confirmed cases for the identification of SD in well-resourced settings. The predictive equation yields odds (*ODD*) that are transformed into probability (*p*) by: *p* = *e^ODD^*/ (*e^ODD^*+1). Patients with *p* greater than 0.0492, 0.0367 should be hospitalized to obtain sensitivities of 0.9, 0.95 and the corresponding specificities of 0.30, 0.25.

|  | **Estimate** | **Odds ratio** | **95% CI** | **p-value** |
| --- | --- | --- | --- | --- |
| Intercept | -1.64 | - | - | - |
| **Gender (female)** | 1.30 | 3.68 | 2.66-5.12 | 0.00 |
| **Fever duration (days)** | -0.21 | 0.81 | 0.72-0.91 | 0.00 |
| **Fever on admission** | 0.88 | 2.42 | 1.69-3.51 | 0.00 |
| **Lymphocyte count** | -0.02 | 0.98 | 0.96-0.99 | 0.00 |
| **Breathlessness** | 1.13 | 3.09 | 1.48-6.11 | 0.00 |
| **Rash** | -0.41 | 0.67 | 0.46-0.95 | 0.02 |
| **Vomiting** | 0.43 | 1.54 | 1.12-2.14 | 0.01 |
| **Abdominal distension** | 1.57 | 4.82 | 0.99-18.37 | 0.03 |
| Lung crackles | -1.73 | 0.18 | 0.01-0.93 | 0.10 |
