# Supplementary material for: Predictive Tools for Severe Dengue Conforming to World Health Organization 2009 Criteria
Source: PLoS Negl Trop Dis. 2014 Jul 10;8(7):e2972. doi: 10.1371/journal.pntd.0002972 (PMC4091876; doi:10.1371/journal.pntd.0002972)
Supplement: Table S3 — Results of the GLM fitting to PCR and serology confirmed cases for the identification of SD without laboratory information. (DOCX) [file pntd.0002972.s003.docx]

Table S3. Results of the GLM fitting to PCR and serology confirmed cases for the identification of SD without laboratory information. The predictive equation yields odds (*ODD*) that are transformed into probability (*p*) by: *p* = *e^ODD^*/ (*e^ODD^*+1). Patients with *p* greater than 0.04783, 0.0427 should be hospitalized to obtain sensitivities of 0.9, 0.95 and the corresponding specificities of 0.30, 0.30.

|  | **Estimate** | **Odds ratio** | **95% CI** | **p-value** |
| --- | --- | --- | --- | --- |
| **Intercept** | -16.90 | - | - | - |
| **Female** | 1.25 | 3.49 | 2.52-4.84 | 0.00 |
| **Fever duration (days)** | -0.23 | 0.80 | 0.71-0.89 | 0.00 |
| **Fever on admission** | 0.52 | 1.68 | 1.05-2.71 | 0.03 |
| **Maximum temperature** | 0.39 | 1.48 | 1.17-1.88 | 0.00 |
| **Breathlessness** | 0.99 | 2.69 | 1.30-5.26 | 0.01 |
| **Rash** | -0.43 | 0.65 | 0.46-0.93 | 0.02 |
| **Vomiting** | 0.44 | 1.55 | 1.12-2.14 | 0.01 |
| **Abdominal distension** | 1.37 | 3.94 | 0.84-14.12 | 0.05 |
