# Supplementary material for: Predictive Tools for Severe Dengue Conforming to World Health Organization 2009 Criteria
Source: PLoS Negl Trop Dis. 2014 Jul 10;8(7):e2972. doi: 10.1371/journal.pntd.0002972 (PMC4091876; doi:10.1371/journal.pntd.0002972)
Supplement: Table S4 — Results of the GLM fitting to PCR confirmed cases for the prediction of SD due to severe hemorrhage. (DOCX) [file pntd.0002972.s004.docx]

Table S4. Results of the GLM fitting to PCR confirmed cases for the prediction of SD due to severe hemorrhage. The predictive equation yields odds (*ODD*) that are transformed into probability (*p*) by: *p* = *e^ODD^*/ (*e^ODD^*+1). Patients with *p* greater than 0.0285, 0.0158 h sensitivities of 0.9, 0.95 and the corresponding specificities of 0.47, 0.35.

|  | **Estimate** | **Odds ratio** | **95% CI** | **p-value** |
| --- | --- | --- | --- | --- |
| **Intercept** | 3.51 | - | - | - |
| **Age (years)** | -0.07 | 0.93 | 0.90-0.96 | 0.00 |
| **Hematocrit change ≥20% platelet count decrease** | 1.88 | 6.52 | 1.53-24.11 | 0.01 |
| **Serum urea, mmol/l** | 0.30 | 1.35 | 1.00-1.79 | 0.04 |
| **Gender female** | 2.19 | 8.93 | 3.26-26.56 | 0.00 |
| **Hemoglobin count** | -0.41 | 0.67 | 0.48-0.92 | 0.01 |
| Eye pain | -16.93 | 0.00 | 0.00->1000 | 0.99 |
