# Supplementary material for: Predictive Tools for Severe Dengue Conforming to World Health Organization 2009 Criteria
Source: PLoS Negl Trop Dis. 2014 Jul 10;8(7):e2972. doi: 10.1371/journal.pntd.0002972 (PMC4091876; doi:10.1371/journal.pntd.0002972)
Supplement: Table S5 — Results of the GLM fitting to PCR confirmed cases for the prediction of SD development due to severe plasma leakage. (DOCX) [file pntd.0002972.s005.docx]

Table S5. Results of the GLM fitting to PCR confirmed cases for the prediction of SD development due to severe plasma leakage. The predictive equation yields odds (*ODD*) that are transformed into probability (*p*) by: *p* = *e^ODD^*/ (*e^ODD^*+1). Patients with *p* greater than 0.0336, 0.0295 have high risk of developing plasma leakage with sensitivities of 0.9, 0.95 and the corresponding specificities of 0.20, 0.14.

|  | **Estimate** | **Odds ratio** | **95% CI** | **p-value** |
| --- | --- | --- | --- | --- |
| (Intercept) | -2.62 | - | - | - |
| **Hematocrit change ≥20% platelet count decrease** | -15.12 | 0.00 | 0.00-14973.94 | 0.98 |
| **Hypoproteinaemia** | 0.68 | 1.96 | 1.10-3.54 | 0.02 |
| Female | 0.76 | 2.14 | 1.17-3.85 | 0.01 |
| **Fever duration (days)** | -0.39 | 0.68 | 0.52-0.87 | 0.00 |
| **Fever on admission** | 1.19 | 3.29 | 1.40-9.36 | 0.01 |
| **Vomiting** | 0.78 | 2.17 | 1.21-3.99 | 0.01 |
| **Abdominal distension** | 4.29 | 72.98 | 4.31-2199.64 | 0.00 |
